# Polysaccharides influence human health *via* microbiota-dependent and -independent pathways

**DOI:** 10.3389/fnut.2022.1030063

**Published:** 2022-11-09

**Authors:** Liping Gan, Jinrong Wang, Yuming Guo

**Affiliations:** ^1^School of Bioengineering, Henan University of Technology, Zhengzhou, China; ^2^State Key Laboratory of Animal Nutrition, College of Animal Science and Technology, China Agricultural University, Beijing, China

**Keywords:** polysaccharide, microbiota, gut health, metabolic disease, biological activity

## Abstract

Polysaccharides are the most diverse molecules and can be extracted from abundant edible materials. Increasing research has been conducted to clarify the structure and composition of polysaccharides obtained from different materials and their effects on human health. Humans can only directly assimilate very limited polysaccharides, most of which are conveyed to the distal gut and fermented by intestinal microbiota. Therefore, the main mechanism underlying the bioactive effects of polysaccharides on human health involves the interaction between polysaccharides and microbiota. Recently, interest in the role of polysaccharides in gut health, obesity, and related disorders has increased due to the wide range of valuable biological activities of polysaccharides. The known roles include mechanisms that are microbiota-dependent and involve microbiota-derived metabolites and mechanisms that are microbiota-independent. In this review, we discuss the role of polysaccharides in gut health and metabolic diseases and the underlying mechanisms. The findings in this review provide information on functional polysaccharides in edible materials and facilitate dietary recommendations for people with health issues. To uncover the effects of polysaccharides on human health, more clinical trials should be conducted to confirm the therapeutic effects on gut and metabolic disease. Greater attention should be directed toward polysaccharide extraction from by-products or metabolites derived from food processing that are unsuitable for direct consumption, rather than extracting them from edible materials. In this review, we advanced the understanding of the structure and composition of polysaccharides, the mutualistic role of gut microbes, the metabolites from microbiota-fermenting polysaccharides, and the subsequent outcomes in human health and disease. The findings provide insight into the proper application of polysaccharides in improving human health.

## Introduction

**FIGURE 1:**
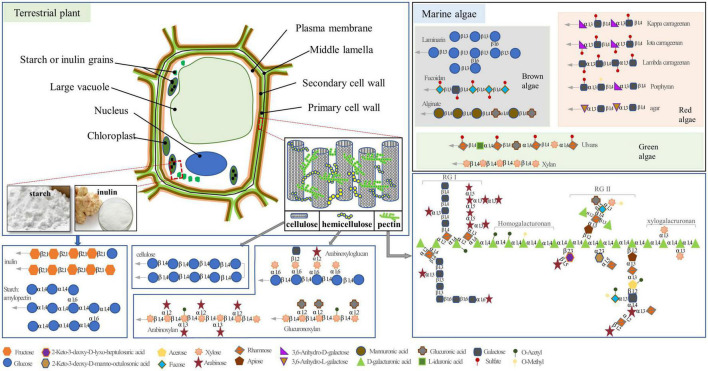
The structure of polysaccharides in plant and marine algae. The gray arrows indicate the possibility of extended polymer length.

Polysaccharides, which are composed of more than 10 monosaccharide units connected by glycosidic linkages, are the most abundant types of carbohydrates and are present in various living organisms, including plants, fungi, and marine algae. Depending on their composition of monosaccharides, polysaccharides are classified as either homopolysaccharides, which comprise only one type of monosaccharide (e.g., starch), or heteropolysaccharides, which are composed of two or more different monomeric units (e.g., pectin). Polysaccharides can serve as reserve carbohydrates and/or structural components that contribute to complex physiological processes in plants and other organisms (1). The reserve polysaccharides primally exist in the cytoplasm, whereas the structural polysaccharides are mainly stored in the primary and secondary cell walls. Both serve as carbohydrate sources, provide fibers in human and animal diets, and affect physical function and health (Figure 1) (2).

Polysaccharides are primarily consumed by oral administration and pass through the intestines for further utilization; therefore, polysaccharides have great biological benefits for bowel health (3). Humans and animals can directly process only simple sugars and a certain type of starch; thus, a large portion of polysaccharides (e.g., fiber) reaches the hindgut intact and is fermented by the intestinal microbiota. The microbiota and their derived metabolites have a great impact on human health and physiology (4). Therefore, considerable research has focused on the interaction between polysaccharides and intestinal microbiota as well as on shaping the structure of gut microbiota to determine polysaccharides’ effects on human health (5). Dietary fiber deficiency changes the gut microbiota and leads to gut dysbiosis, which occurs in various diseases, especially metabolic diseases (6). The increased incidence of insulin resistance, obesity, and other metabolic disease is partly due to increased systemic and tissue inflammation caused by increased systemic levels of bacterial endotoxins and DNA (7). Therefore, improving gut health through polysaccharide intervention, which can manipulate gut microbiota, can influence metabolic disease (8).

Furthermore, the influence of polysaccharides on gut health and metabolic diseases is not limited to mechanisms linked to the intestinal microbiota. Some *in vitro* studies have shown that polysaccharides can directly modulate the health of humans. Astragalus polysaccharides protected bladder epithelial cells against *Escherichia coli* infection by upregulating TLR4 expression and subsequently increased the secretion of IL-6 and IL-8 (9). Polysaccharides can activate the B-cell TLR4/TLR2-p38 MAPK signaling pathway to enhance immune response (10). In addition, some polysaccharides, such as the pectin-type polysaccharides from *Smilax china* L., can be absorbed in the small intestine and are distributed in the liver and kidney (11). Oral absorption constitutes the basis of the direct effect of polysaccharides on human health. The widespread distribution and fundamental function of polysaccharides in plants as well as the extraction of different polysaccharides from various organisms and their positive effects on the health of humans and animals have been reported (12). However, it is unclear whether polysaccharides from different organisms have similar effects on animals and humans or if it is necessary to extract polysaccharides from various plants or other organisms even when their polysaccharide concentration is low. Therefore, this review focuses on how polysaccharides from terrestrial plants, fungi, and marine algae influence human health, especially gut health and metabolic disease. Additionally, it aims to identify the underlying mechanisms of bioactive polysaccharides in gut health and metabolic disease to provide insight for further research and application of polysaccharides in human and animal health.

## Statistical review of the effects of polysaccharides on health

**FIGURE 2:**
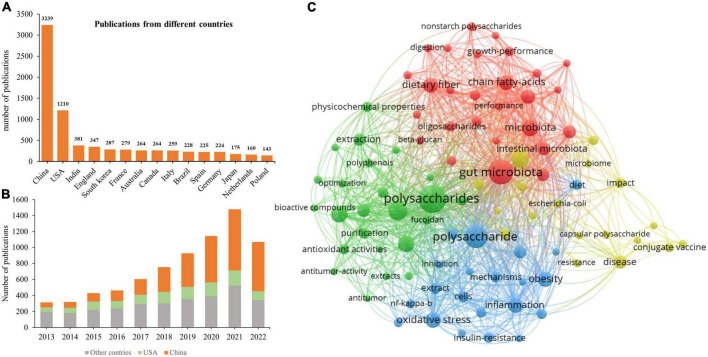
Publication analysis of polysaccharides in the last 10 years. **(A)** Total publications related to polysaccharides and human health from 2013 to 2022. **(B)** The yearly output from different countries. **(C)** Network visualization of terms associated with polysaccharides and human health.

Research on the influence of polysaccharides on human and animal health published during 2013–2022 was ascertained using VOSViewer, and the terms “polysaccharides” and “health or gut health or microbiota or obesity or type 2 diabetes or non-alcoholic fatty liver disease” were searched in the Web of Science. A total of 7,497 records, including 1,590 review articles, 5,799 articles, and 459 other types of documents, were downloaded from the SSCI database of Web of Science. The yearly publication of related topics has been continually increasing (Figure 2), depicting the increased interest in research on the effects of polysaccharides on health. Of note, the number of publications in 2022 (Figure 2) represents those published in the first three quarters of the year, as the search in Web of Science was conducted on 10 September 2022. Therefore, the number of publications on “polysaccharides” and “health” will likely to exceed 1,500 in 2022. Among the countries that have published more than 130 related articles, both China and the USA have the most publications (3,239 and 1,210, respectively; Figure 2A). Furthermore, the number of publications from China has increased dramatically since 2017 (Figure 2B). The increased number of publications on polysaccharides and its effects on human and animal health may be attributable to the Chinese medicinal processing activities as water extraction is the main method that is used to prepare Chinese medicines, and this method is similar to the procedure for the extraction of polysaccharides. The major keywords that were associated with the search terms which appeared more than 100 times were summarized (Figure 2C), and the top 15 keywords are listed in Table 1. Unsurprisingly, except for “polysaccharides,” “intestinal microbiota” was the most frequently identified keyword in the publications. Intestinal microbiotas play a vital role in the digestion of polysaccharides and exert functions on the health of humans and animals. Furthermore, “antioxidant ability” appeared frequently in the downloaded publications, thereby indicating the biofunctions of polysaccharides as antioxidants. The “sulfated polysaccharides” and “fucoidan” that were found in various species of brown algae have increasingly received attention for their marked antioxidant ability. Moreover, from the occurrences of “extraction,” “structural characterization,” and “purification,” we can infer that, with the development of sequencing and other technologies, scientists have become more interested in obtaining pure polysaccharides to clarify their structural characteristics and functions.

**TABLE 1:** The top 15 highest occurrences of keywords.

Items	Occurrences	Total link strength		Occurrences	Total link strength
Keywords					
Polysaccharides	3748	11964	Inflammation	415	1602
Gut microbiota	2526	9027	Fermentation	378	1650
Antioxidant activity	1278	4151	Metabolism	336	1246
*In vitro*	673	2102	Disease	314	845
Health	580	1948	Structural characterization	313	1082
Extraction	527	1759	Expression	306	917
Chain fatty-acids	418	1800	Purification	247	899
Obesity	416	1529			

## Interaction between polysaccharides and microbiota

The gastrointestinal tract houses several trillion microbial cells which are strongly associated with human health. Carbohydrates are the main source of energy and nutrients for intestinal microbiota and thus influence microbial composition through the modulation of specific species and their derived metabolites (13). Moreover, the microbiota possesses a larger repertoire of degradative enzymes and is adept at foraging glycans and polysaccharides that are derived from plants, animals, and other sources (14). The mutual dependence between polysaccharides and gut microbiota constitutes an important basis for the participation of polysaccharides in a diverse array of physiological processes in humans.

### Polysaccharides degradation by microbiota

The huge diversity of polysaccharides has partly resulted from the various component sugar substituents and their linkage patterns, which can be branched at different positions on a single substituent by α- or β-glycosides (15). In addition, polysaccharides can be covalently coupled to other molecules, such as protein, lipids, and even RNA (16), and thereby adopt a secondary structure. At the same time, some studies have revealed the three-dimensional molecular conformation of polysaccharides, such as polysaccharides from *Laminaria japonica* (17), which inevitably adds complexity to the polysaccharides.

In general, the more complex the polysaccharides are, the greater the number of enzymes that are required in the breakdown process. However, for humans, only 17 enzymes are encoded for the digestion of food glycans, specifically for a certain type of starch (18), whereas gut bacteria can produce hundreds of enzymes with catalytic specificities that range well beyond that of starch (15, 19). The carbohydrate-active enzymes (CAZymes), which are encoded by intestinal microbiota, are required to break down the glycoconjugates and polysaccharides to release fermentable monosaccharides that can be used as an energy source by intestinal cells and/or bacteria. Glycoside hydrolases (GHs) and polysaccharide lyases (PLs) are two main types of CAZymes that cleave glycosidic bonds between carbohydrates and between a carbohydrate and a non-carbohydrate moiety (18). The animal gut harbors trillions of microbes, of which Firmicutes and Bacteroidetes are the most commonly represented phyla. The Bacteroidetes encode more CAZymes than other phyla (18). *Bacteroides thetaiotaomicron*, a dominant member of human distal gut microbiota, contains more than 261 GHs and PLs (20). Furthermore, the comparative genomic analysis revealed that fully sequenced intestinal Bacteroidetes contain genes that encode sulfatases and the related active enzymes, which are crucial for fermenting sulfated polysaccharides, such as mucin and glycosaminoglycans in mucus, as well as fucoidans in brown seaweeds and carrageenan in red seaweeds (21, 22). With the capacity to utilize an extensive array of dietary and host-derived polysaccharides, the Bacteroidetes are considered glycan-degrading generalists. However, Firmicutes and Actinobacteria appear more specialized with a preference for the reserve polysaccharides of plants (23).

Different phyla have different fermentation mechanisms for processing polysaccharides. The gram-negative Bacteroidetes pack their diverse array of CAZymes into discrete polysaccharides utilization loci (PUL) gene clusters, which have been identified in all intestinal Bacteroidetes and encode substantial numbers of surface proteins that are required for the utilization of polysaccharides. Therefore, the polysaccharides targeted by Bacteroidetes require extracellular hydrolysis before being transported into the cell. The well-studied starch utilization system (Sus) is the first PUL that was described for starch processing in *B*. *thetaiotaomicron* (24). However, in contrast to the Bacteroidetes, the gram-positive Firmicutes and Actinobacteria depend more on a diverse array of transporters, such as ABC-transport systems, to import smaller sugars for intracellular processing, which provides an important competitive advantage against the predominant Bacteroidetes (25). The mechanisms of polysaccharide degradation that use either the PUL or Sus system by Bacteroidetes and the ABC system by Firmicutes and Actinobacteria have been described previously (26) and are not covered in depth here. Overall, the microbiota plays a critical role in the host’s digestion of polysaccharides.

### Influence of polysaccharides on microbiota

The exceptional diversity of dietary polysaccharides has a profound influence on the composition and structure of intestinal microbiota (27). Different microbial species have different preferences for glycans, which determine the structure and monosaccharide composition of polysaccharides and have a great impact on intestinal microbiota. Wu et al. (28) reported that okra pectic-polysaccharides with different structures selectively changed the composition of intestinal microbiota (27). *Enteromorpha* polysaccharide enriched the abundance of *Bacteroides*, which helps to break down the polysaccharides (29). At the same time, several studies that focused on the capacity of gut bacteria to catabolize marine algal polysaccharides, such as porphyran and agarose, have revealed the geographic distribution of intestinal microbiota (30–32). *B. plebeius*, which contains genes that encode porphyranases and agaroses, has been isolated from Japanese individuals whose diet typically includes seaweed. However, the gut metagenome analyses from North American individuals showed the absence of porphyranases and agaroses (31). Furthermore, a study of *Desulfobulbus* and *Methanosarcina* indicated that the spatial distribution of microbial communities significantly correlated with geographic distance (32). The abovementioned studies indicated that the sources of polysaccharides directly influence the composition of intestinal microbiota. Moreover, the inclusion of pea fiber in the diet of gnotobiotic mice that were cloned with a defined consortium of human-gut-derived bacteria significantly increased the abundance of *B. thetaiotaomicron*. In addition, the richness of *B. cacccae* in the model revealed the pronounced effects of high-molecular weight inulin on the composition of the microbiota (33). Polysaccharides can directly encourage the expansion of certain bacterial species by serving as nutrient sources for their growth. Another study that involved the incubation of different human gut-derived bacteria with different glycans *in vitro* showed that some species and strains from *Bacteroides* and *Parabacteroides* exhibited the ability to bind one or more specific glycans, thereby indicating that different glycans are responsible for the expansion of different bacterial species or strains (34). Furthermore, microbiota that has limited metabolic capacities for processing complex polysaccharides must rely on other organisms that are capable of fermenting polysaccharides through microbe–microbe interactions, such as commensalism, mutualism, and competition (26, 33, 35, 36). Therefore, many types of complex polysaccharides help to confer additional diversity to the gut microbiota partly through the interactions among microbes.

Different types of polysaccharides enable rational manipulation of the microbiota based on the species’ metabolic capacity. The CAZymes (e.g., extracellular β-2,6 endo-fructanase) that are encoded by intestinal bacteria enable the metabolic processing of β-2,6-linked fructan levan. Therefore, dietary involvement of β-2,6-linked fructan levan enriches the abundance of *B. thetaiotaomicron* (37). Genome analysis coupled with efforts to culture human gut microorganisms is constantly aiding the elucidation of the mechanisms underlying mutualistic behavior, which has long been attributed to human gut microbes in the processing of dietary fiber polysaccharides (15, 23, 34, 38). The interaction between microbiota, polysaccharides, and their subsequent metabolites are highly correlated with human health and physiological process.

## Polysaccharides play vital roles in the physiological status of humans

Dietary polysaccharides have diverse, crucial influences on human health. Interactions with microbiota partly explain the underlying mechanisms as polysaccharides are predominantly administered *via* the oral route, and therefore, exert functions for improving human health through their absorption. Due to the lack of methods and technologies to detect polysaccharides, some researchers consider that polysaccharides have poor intestinal absorption after oral administration. However, with improved detection technology, studies have found that after oral administration, polysaccharides can be absorbed into the circulatory system even if they have high molecular weight and complicated structures (11, 39, 40). Moreover, the oral absorption mechanisms of polysaccharides and the factors influencing them are well-reviewed by Zheng et al. (41) and are accordingly not covered in depth here. Overall, direct gut absorption and the interaction with intestinal microbiota are key aspects for understanding the mechanisms of polysaccharide function in human intestinal and metabolic health.

### Polysaccharides influence intestinal health

A functional intestine and an intact intestinal barrier, which permit nutrient transport from the lumen into the blood and simultaneously restrict the passage of potentially harmful microorganisms and toxins, constitute an integral regulator of human health (7, 42). Observational findings that have been accumulated during the last 10 years suggest that polysaccharides have profound biological benefits for bowel health, including anti-inflammation, gut epithelial barrier protection, and immune modulation through both microbiota-dependent and -independent mechanisms (3, 12). Most polysaccharides pass through the small intestine intact and can successfully reach the large bowel, where they can be either fermented by the microbiota or excreted in the stool. Due to their capacity for water retention, polysaccharides in the large bowel could attract water and add bulk to the digesta which increases intestinal peristalsis and softens the stool, thus diluting toxin concentrations, increasing the frequency of defecation, and preventing constipation and its associated problems, such as hemorrhoids (3, 43, 44). Moreover, dietary ingestion of high concentrations of non-starch polysaccharides (NSP) is associated with increased stool weight and a decreased risk of bowel cancer (45). In addition, polysaccharides enhance bowel health by promoting the immune system and reducing inflammation. Polysaccharides from astragalus that mainly contained rhamnose, glucose, galactose, and arabinose ameliorated dextran sulfate sodium (DSS)-induced colitis and increased the colon length by inhibiting NF-κB activation, and thus downregulating TNF-α, IL-1β, and IL-6 expression and subsequently reducing proinflammatory responses (46). Similarly, *Scutellaria baicalensis* Georgi polysaccharides, which are mainly composed of mannose, ribose, glucuronic acid, glucose, xylose, and arabinose, suppressed DSS-induced colitis through inhibition of NF-κB and NLRP3 inflammasome activation, and thereby decreasing pro-inflammatory cytokines secretion in mice and macrophages (47). There is increasing evidence that Peyer’s patches hold the key to how polysaccharides enhance intestinal immune status. Polysaccharides from molokhia (*Corchorus olitorius* L.) leaves could increase bone marrow cell proliferation as well as immunoglobulin A and cytokine production *via* Peyer’s patches (48), which is consistent with the hypothesis of Han (49) who states that polysaccharides could enter Peyer’s patches to trigger immune responses even without entering the blood circulation. Moreover, polysaccharides from *Coptis chinensis* Franch. (50), *Atractylodes lancea* (51), and *Lavandula angustifolia* Mill. (52) could be taken up by Peyer’s patches and stimulate the immune cells inside it to regulate cytokine secretion. Therefore, polysaccharides can exert immune-enhancing functions without absorption into the bloodstream, which benefits gut health by improving the immune status of the gut. Furthermore, polysaccharides, such as α-D-glucan, could enhance the intestinal barrier function by increasing the expression of tight junction proteins (53, 54).

**FIGURE 3:**
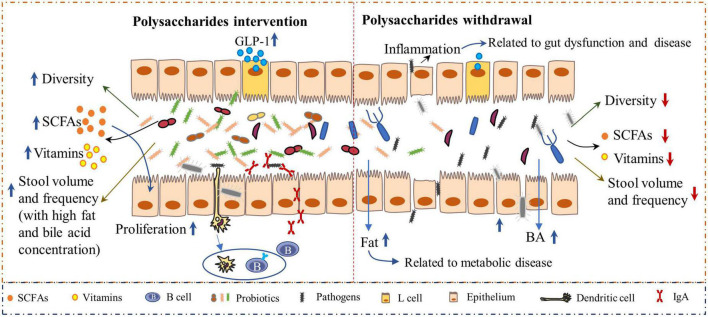
The inclusion of polysaccharides has a profound impact on gut health. Polysaccharide-based interventions increased microbiota-derived metabolites, such as short-chain fatty acids (SCFA) and vitamins **(Left)**. SCFAs can bind to receptors on L cells and subsequently induce the secretion of glucagon-like peptide 1 (GLP-1), which can affect energy expenditure (132). Polysaccharides are highly associated with increased stool volume, frequency, and fat and bile acid concentrations (45, 46, 133), which reinforce gut health. Moreover, the intestinal immune system is enhanced by polysaccharides, as indicated by the increased secretion of immunoglobulin A (sIgA) levels. However, when the diet contains very low concentrations of polysaccharides, the balance between the gut microbiota and immunity will be disrupted **(Right)**, resulting in decreased diversity of microbiota with an increased abundance of pathogens, which elicit gut inflammation and subsequent bowel disease.

Additionally, the interaction of polysaccharides and intestinal microbiota plays a crucial role in gut health. A deficiency of dietary polysaccharides leads to gut dysbiosis. As the microbiota mostly relies on polysaccharides as a nutrient source, the absence of these nutrients in the diet forces the microbiota to transition toward the use of indigenous host glycans, which causes the expansion of pathogenic organisms and decreased abundance of probiotics and the linked metabolites. Evidence has revealed that the microbiota can erode the colonic mucus layer in the absence of dietary polysaccharides, thus accelerating enteric pathogen invasion and intestinal disease progression when challenged with the pathogen *Citrobacter rodentium* (15, 55). Low concentrations of dietary polysaccharides induced inflammation and increased intestinal permeability that led to increased pathogen invasion into other tissues, which is highly associated with the onset of obesity and other metabolic diseases (56) (Figure 3). Comparatively, the dietary inclusion of polysaccharides is important for supporting the function and stability of gut microbiota and, eventually, for maintaining gut health. Polysaccharides derived from *Lentinula edodes* encouraged the expansion of *B. acidifaciens* (57). In addition, polysaccharides from *Flammulina velutipes* improved colitis by shaping the structure of the colonic microbiota and inflammatory responses. Bacteria-derived polysaccharides, including glucorhamnan, which are synthesized and secreted by *Ruminococcus gnavus*, influence intestinal health *via* the regulation of intestinal inflammatory states (58). Furthermore, the microbiota-derived metabolites, such as short-chain fatty acids (SCFAs) (59), enhanced the intestinal fermentation of diverse polysaccharides and have profound effects on bowel health. SCFAs can be used directly as energy sources by colonic epithelial cells, support their proliferation, and enhance the epithelial barrier function (60). Polysaccharides from *Cistanche* (61), *Vigna radiata* L. skin (62), enriched probiotic bacteria and SCFA in the intestine of mice. In addition, both *in vivo* and *in vitro* studies indicated that polysaccharides from soybean or marine algae could enhance the abundance of probiotic bacteria whereas inhibiting pathogens in the intestine (19, 63, 64). Thus, polysaccharides are crucial for intestinal health, which further benefits the health of the body.

### The relationship between polysaccharides and obesity

**FIGURE 4:**
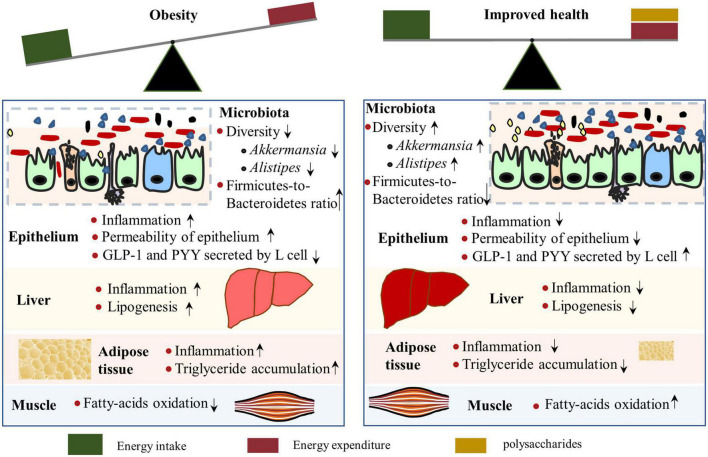
Mechanisms by which polysaccharides alleviate obesity. A polysaccharide-rich diet contributes to the maintenance of a healthy gut and reduces inflammation of the liver and adipose tissue **(Right)**. Intestinal microbiota composition is associated with obesity, of which low diversity, reduced abundance of *Akkermansia* and *Alistipes*, and enhanced Firmicutes-to-Bacteroidetes ratio were observed in obese individuals. However, polysaccharide supplementation can reverse the microbiota changes in obese situations, along with increased glucagon-like peptide 1 (GLP-1) levels, which is positively related to energy expenditure.

The prevalence of obesity has been increasing dramatically worldwide, and the progression and maintenance of obesity include genetic and environmental factors, diet (e.g., high availability of high-energy foods with less dietary fiber), and lifestyle (e.g., sedentary ways of life) that leads to excess peripheral and visceral lipid accumulation (65). Moreover, dysbiosis of intestinal microbiota acts both as a cause and a consequence of obesity (66–68). Notably, obesity is associated with systemic low-grade inflammation and various health issues, such as type 2 diabetes (due to insulin resistance), fatty liver disease, short life expectancy, and so on (69). Therefore, identifying efficient strategies to prevent or ameliorate obesity is important for the health of people who are overweight or obese. Recently, interest in the role of polysaccharides in preventing obesity has increased, and the anti-obesity properties and mechanisms of polysaccharides have been reported by several studies (70–72) (Figure 4).

Most polysaccharides cannot be digested to directly provide energy to animals. Therefore, the dietary inclusion of polysaccharides could reduce calorie intake. Moreover, due to their complex special structure, polysaccharides are characterized by great fat-binding capacities, which leads to the increased excretion of dietary or endogenous fatty acids (73). Polysaccharides can bind bile acids in the intestine to enhance its excretion, thus enabling new bile acid synthesis in the liver and consuming more cholesterol (74). Consistent results were obtained in research on xyloglucan and inulin supplementation, which increased the fecal total bile acid concentration (75). Decreasing the energy intake as well as increasing fatty acids and cholesterol excretion is of great importance for decreasing lipid accumulation, and thus could benefit overweight individuals. Besides this, enhancing energy expenditure is another mode of action that actualizes the anti-obesity property of polysaccharides. *Lyophyllum decastes* polysaccharides enhance energy expenditure in diet-induced obese mice, which might be due to the upregulation of the secondary bile acids-activated TGR5 pathway (74). Furthermore, the enhanced brown tissue activity by polysaccharides (74, 76) could explain the energy expenditure property of polysaccharides to some extent.

Inhibition of lipogenesis and promotion of lipolysis/fatty acid oxidation are very important to restrict fat accumulation. Peroxisome proliferator-activated receptor gamma (PPARγ) is a transcriptional factor that directs the differentiation of adipocytes, whereas PPARα is a key transcriptional factor for fatty acid oxidation (77). In addition to dietary sources, endogenous fatty acid production from *de novo* lipogenesis in mammalian tissues, including liver, white adipose tissue, and brown adipose tissue, has been identified in both healthy and obese individuals. Polysaccharides inhibit hepatic lipogenesis and lipogenesis in white adipose tissues, (78, 79), mainly through the inhibition of core enzymes, such as acetyl-CoA carboxylase (ACC) and fatty acid synthase (FAS), in the lipogenic process (80). Moreover, PPARγ expression could be inhibited by dietary polysaccharides in the liver and adipose tissues of diet-induced obese mice (81). *In vitro* experiments using 3T3-L1 cells demonstrated the direct inhibition of adipocyte differentiation by quinoa polysaccharide through PPARγ inhibition (79, 82, 83), and activation of the AMPK/PPARα pathway by polysaccharides was observed in obese mice, which implies increased fatty acids oxidation and energy expenditure. Therefore, polysaccharides could prevent obesity and/or ameliorate obesity by inhibiting lipogenesis while enhancing lipolysis. Although polysaccharides with anti-obesity properties have different sources, structure, and composition, they have similar modes of actions in ameliorating diet-induced obesity.

The fundamental influence of polysaccharides on intestinal microbiota explains its primary mechanism in reducing obesity, which has been studied in many research articles (70, 71, 84, 85) and reviews (86, 87). High-weight molecular polysaccharides isolated from *Ganoderma lucidum* reduced body weight and fat accumulation in obese mice by altering the intestinal microbiota composition, as indicated by the decreased Firmicutes-to-Bacteroidetes ratios and improved gut barrier function. Research on HG-type pectin, derived from *Ficus pumila* L. fruits, increased the abundance of *Akkermansia* and *Alistipes* in obese mice. The subsequent metabolites, myristoleic acid, and pentadecanoic acid, are negatively associated with serum lipid concentration and contribute to decreased fat concentration (88). A fucoidan from Sargassum fusiform has similar effects, which restored *Alistipes* abundance (89). The microbiota species enriched by polysaccharides in obese animals correlated with a reduction of obesity, thus providing insights to guide the development of probiotics and functional prebiotics to prevent obesity in clinical practice.

Interestingly, xyloglucan compounded with arabinoxylan or inulin supplementation activated intestinal or hepatic G protein-coupled 5 (TGR5) of mice that were fed a high-fat diet (75). TGR5 signals in enteroendocrine L-cells induce glucagon-like peptide 1 (GLP-1) and peptide YY (PYY) excretion, thereby attenuating food consumption rate, improving liver and pancreatic function, and promoting glucose metabolism, as well as activating TGR5 in adipose and muscle tissues to increase energy expenditure (90). TGR5 activation by polysaccharides prevents diet-induced obesity through attenuation of energy intake and increased energy expenditure. Therefore, dietary inclusion of more of the abovementioned polysaccharides is considered a good strategy to alleviate obesity.

### Polysaccharides and control of type 2 diabetes

Diabetes mellitus comprises a group of metabolic diseases characterized by chronic hyperglycemia, along with many complications, such as diabetic nephropathy and cardiovascular disease. Usually, diabetes can be divided into two main broad categories: type 1 diabetes and type 2 diabetes mellitus (T2DM), which account for the majority (∼90%) of total diabetes prevalence (91, 92). Known as non-insulin-dependent diabetes mellitus, T2DM is largely induced by insulin resistance and dysfunction of insulin-producing β cells, which decreases the tissue sensitivity to insulin and has insufficient biological effects, thereby leading to hyperglycemia (91). However, unlike type 1 diabetes, which is not preventable with the current knowledge, effective approaches are available to prevent T2DM and its complications (93). Increasing evidence has shown that polysaccharides exhibit antidiabetic effects. Considering the growing reports on polysaccharides as therapy for T2DM and their popularity as dietary supplements, this subsection is designed to clarify the various mechanisms of such therapeutic applications.

The application of polysaccharides in the diet- and/or drug-induced T2DM animal models ameliorated glucose tolerance (94), inhibited insulin resistance (95), protected damaged pancreatic islets (96), improved β cell function (95), enhanced lipid metabolism thus increasing insulin sensitivity in the liver (97), and reduced oxidative stress and inflammatory response (98) to relieve T2DM. Polysaccharides from *Anoectochilus roxburghii* could inhibit the key gluconeogenesis enzymes, thereby increasing glucose absorption (99), which explains the function of polysaccharides in decreasing fasting blood glucose levels. *Echinops* spp. polysaccharide B could increase muscle and liver glycogen content (100), which lowers the blood glucose level in T2DM. Polysaccharides from *Sphacelotheca sorghi* (Link) Clint (101) and *Auricularia auricula-judae* (102) enhanced the hepatic health of T2DM by activating the PI3K/Akt signaling pathway. *Echinops* spp. polysaccharide B increased the number of insulin receptors in the liver and muscles, thus decreasing insulin resistance in T2MD (100). Besides their use as a dietary source, polysaccharides can be used to protect insulin that is administered orally. The ability to improve the permeability *via* transcellular and/or paracellular pathways and even selectivity for targeted delivery of insulin through nano- and microencapsulation of polysaccharides is considered an important technological strategy to protect insulin against the harsh conditions of the gastrointestinal tract (103).

In addition to the abovementioned functions, polysaccharides can affect T2DM by influencing the structure of intestinal microbiota and their derived metabolites, the composition of which plays pivotal roles in the pathogenetic process of T2DM (104). Patients with T2DM have increased relative abundances of the phyla Firmicutes and Actinobacteria and decreased relative abundances of Bacteroidetes. Consistently, Lactobacillus and Eubacteria were significantly enriched (104), whereas abundances of Bifidobacterium were decreased in T2DM patients (105). Inulin supplementation increased the abundance of *Bifidobacterium* and increased the integrity of the gut barrier, which was negatively correlated with T2DM (75, 105). *Apocynum venetum* polysaccharides reversed the gut microbiota dysbiosis in diabetic mice by increasing probiotic abundances, such as *Odoribacter*, *Parasutterella*, *Lactobacillus*, and *Akkermansia*, whereas decreasing *Enterococcus* and *Aerococcus* levels, which are correlated with improved liver glycogen contents and reduced insulin resistance (95, 106, 107). Dietary polysaccharides enriched the SCFA-producing strains in the intestine, including *Bifidobacterium* and *Romboutsia*, thus enhancing SCFAs concentrations, inhibiting the growth of other detrimental bacteria, and benefiting T2DM patients (104, 108). The bacteria-derived SCFAs have been shown to decrease proinflammatory cytokines and inhibit lipolysis in adipose, which is responsible for glucose disposal of T2DM patients by regulating free fatty acids in blood (109). Butyrate was reported to improve hepatic fatty acid oxidation and activate the AMPK-acetyl-CoA carboxylase pathway, thereby regulating glucose metabolism and inhibiting insulin resistance in the liver (95, 110). Meanwhile, acetate intervention in obese mice improved the expression of genes involved in oxidative and glucose metabolism and glucose transporter in skeletal muscle, enhancing glucose disposal for which skeletal muscle accounts for 85% of postabsorptive blood glucose (111). Collectively, considering the high price as well as the indistinct safety property of the drug used in T2DM patients currently, polysaccharides with anti-diabetes features can be used as promising ingredients for T2DM patients.

### The role of polysaccharides in non-alcoholic fatty liver disease

**FIGURE 5:**
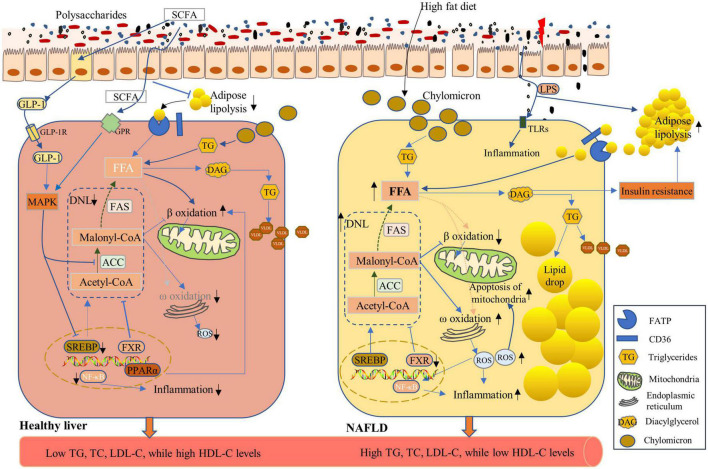
Effects of polysaccharides on the non-alcoholic fatty liver disease (NAFLD). NAFLD is characterized by increased lipid accumulation within hepatocytes, mainly through the uptake of chylomicron processed from dietary fat, circulating free fatty acids (FFA) from lipolysis of adipose tissues, and elevated *de novo* lipogenesis (DNL) (119, 123) **(Right Panel)**, leading to high levels of triglycerides (TG), total cholesterol (TC), and low-density lipoprotein-cholesterol (LDL-C), as well as low levels of high-density lipoprotein-cholesterol (HDL-C) in the serum. High-fat diet induces high levels of chylomicron storage in the hepatocytes, which contributed to high FFA levels in hepatocytes. An intermediate metabolite in triglyceride synthesis, diacylglycerol (DAG), induces insulin resistance, which further enhances the lipolysis of adipose tissues and the subsequent high FFA concentrations. FA synthesis is catalyzed by acetyl-CoA carboxylase (ACC) and fatty acid synthase (FAS), and their expression can be induced by sterol-response-binding-protein-1c (SREBP-1c) and be inhibited by farnesoid X receptor (FXR). Under NAFLD conditions, SREBP-1c expression was enhanced with enhanced DNL **(Right Panel)**. Polysaccharide application enhanced the expression of FXR and reduced DNL. Lipid accumulation within hepatocyte was limited, and FA oxidation was enhanced via improved β-oxidation and GLP-1 function to ensure healthy hepatic lipid metabolism **(Left Panel)**.

Non-alcoholic fatty liver disease (NAFLD) is a chronic liver disease characterized by excess triglyceride accumulation in hepatocytes due to both increased inflow of free fatty acids and *de novo* hepatic lipogenesis, which affects a high proportion of the world’s population (112). Mechanistic insights into fat accumulation, subsequent hepatocyte injury, and the roles of the immune system and gut microbiome are unfolding (113). The inflow of lipids accumulated in livers mainly originates from three processes namely, *de novo* lipogenesis (DNL), dietary sources, and circulating esterified-fatty acids. Moreover, approximately 40% of the lipids derive from DNL and dietary sugars and fats, whereas the remaining 60% arise from lipolysis of dysfunctional adipose tissues (114, 115). Furthermore, the diacylglycerol intermediates, accumulated during the above-described process, impair hepatic insulin signaling by activating protein kinase Cε (PKCε) (116). Hepatocyte insulin resistance promotes hyperglycemia and enhances more compensatory insulin production, which prompts DNL by activation of carbohydrate-response element binding protein (ChREBP) and sterol regulatory element binding protein-1c (SREBP-1c) (113). ChREBP and SREBP-1c synergistically induce FAS and ACC expression, which catalyzes fatty acid synthesis, and are complexly regulated by various nuclear receptors, such as PPARα and farnesoid X receptor (FXR) (117–119) (Figure 5). Reduced hepatic fatty acid oxidation was reported among the pathophysiological changes of NAFLD (120). Accumulated fatty acids inside hepatocytes impose a strain on mitochondria, leading to the dysfunction of mitochondria and the production of ROS. The ROS and subsequent activation of Jun N-terminal kinase (JNK) in turn result in mitochondrial damage, which adds to the stress on the endoplasmic reticulum and further inhibits β oxidation of fatty acids. Moreover, hepatic inflammation, which is triggered by fatty acids, bacterial endotoxins, and ROS, exacerbates hepatocyte damage (113, 119, 121).

To date, there are no effective medical interventions to completely reverse NAFLD other than diet/lifestyle modification. However, polysaccharides that target the hepatocytic DNL, inflammation of the liver, and intestinal microbiota currently have been under investigation to develop promising pharmacological therapies for the treatment of NAFLD. *Ginkgo biloba* leaf polysaccharides (GBLP) are mainly composed of galactose (32.21%), mannose (20.82%), glucose (9.39%), arabinose (6.71%), rhamnose (14.76%), and galacturonic acid (16.11%), which markedly reduced the serum levels of TC, triglycerides, LDL-C, and free fatty acids and significantly increased HDL-C concentrations in NAFLD rats induced by a high-fat diet. Levels of hepatic triglycerides and lipids decreased after GBLP administration in NAFLD rats (122). As increased DNL is a distinct characteristic of NAFLD (123), it is important to impede the process by using functional ingredients. Guar gum supplementation in chicken diet markedly increased SCFA concentrations, leading to increased GLP-1 levels, activation of mitogen-activated protein kinase (MAPK) pathways in hepatocytes, and subsequent suppression of lipid accumulation in hepatocytes by inhibiting SREBP1 and ACC activities (124). Chicory polysaccharides inhibited DNL through the inhibition of genes related to DNL in hepatocytes, whereas the β-oxidation and anti-inflammatory factors were enhanced in NAFLD rats (125). Based on the serum metabolomic analysis, chicory polysaccharides inhibited fatty acid biosynthesis and enhanced β oxidation of very long-chain fatty acids, which implies the probable mechanisms for alleviating NAFLD (126). *Ganoderma amboinense* polysaccharides enhance hepatic fat transport and mitochondrial function in NAFLD mice. MDG-1, an insulin-like β-fructan polysaccharide extracted from Ophiopogon japonicus, decreased the activity of PPARγ and upregulated the expression and phosphorylation of AMPK, SREBP-1c, and ACC-1, thus improving lipid metabolism in high-fat diet mice and reducing the pathogenesis of NAFLD (127). Targeting intestinal microbiota is another strategy to prevent NAFLD. MDG significantly increased the diversity of microbiota, of which *Akkermansia muciniphila* was highly abundant following MDG intervention in NAFLD mice (128). However, most trials evaluating the function of polysaccharides were conducted in animal or cell models and further research is needed to identify whether polysaccharides have therapeutic effects on NAFLD patients, and more clinical trials should be conducted.

## Limitations and perspectives

Due to the natural source and low toxicity of polysaccharides, considerable efforts have been focused on discovering polysaccharides that can be used as novel therapeutics in various diseases (129). Polysaccharides can be used as carriers to protect some labile drugs and facilitate their survival in hostile gastrointestinal tract environment (103). Interestingly, most polysaccharides exhibit positive effects on human health although they have different compositions and structures. Moreover, publications on polysaccharides are steadily increasing for various reasons. First, as polysaccharides exist in almost all living systems, it is reasonable to infer that thousands of different polysaccharides can be extracted. Furthermore, the extracted polysaccharides usually are not composed of one pure substrate but comprise a mixture of a series or different kinds of polysaccharides with diverse chain lengths and dissimilar branches or linkages. Therefore, the extraction conditions will highly influence the composition and the structure of the polysaccharides, which might induce different consequences when applied under different conditions. However, as the functional ingredients can be directly obtained from the diet, the extraction of polysaccharides from edible plant or organisms that needs considerable energy expenditure is not recommended. Furthermore, Han et al. (130) reported that the functional ingredients of N-methylserotonin from orange fibers by-products were released by intestinal microbiota, which might be disposed of in the extraction process. Therefore, additional efforts are needed to identify functional polysaccharides from non-edible dietary by-products.

Additionally, the polysaccharide-interaction-based approach to promote health is unlikely to elicit consistent effects across individuals (131). The large molecular weight and complex structure of polysaccharides limit their usage in tissues other than the intestine, as the majority of polysaccharides cannot be digested in the small intestine or absorbed by the intestinal epithelium. Most of the functions of polysaccharides in other tissues are mediated through metabolites obtained *via* fermentation by microbiota. However, the gut microbes varied among different individuals, which explains why the interindividual variation in the gut microbiome is usually linked to differential effects of polysaccharides on the host metabolic phenotypes. Experiments for detecting the function of polysaccharides in different health conditions are warranted, and more clinical trials should be conducted to enable the application of polysaccharides as therapeutic drugs. However, the development of more efficient and economic approaches for the preparation and modification of polysaccharides and elucidation of the structure-activity relationship remain as significant challenges.

## Author contributions

LG, JW, and YG wrote the manuscript. JW had primary responsibility for final content. All authors read and approved the final manuscript.
